# A GIS-based land suitability model for agricultural tractors in CALABARZON Region, Philippines

**DOI:** 10.1038/s41598-023-45071-w

**Published:** 2023-10-25

**Authors:** Rossana Marie Amongo, Ronaldo Saludes, Ralph Kristoffer Gallegos, Patrick Lemuel Relativo, Ria Salustia Duminding, Adrian Daniel Pantano, Julius John Paul Cunan, Gherlee Nelle Lalap-Borja

**Affiliations:** IABE, CEAT, UPLB, Los Baños, Laguna Philippines

**Keywords:** Sustainability, Engineering

## Abstract

Agricultural machinery distribution programs are key components of the Philippine government to strengthen its economic productivity. However, concerns were reported that some of the distributed machines were under-utilized or unutilized in their respective farms. This study is focused on the development of a GIS-based model of determining suitable lowland rice areas for two-wheel and four-wheel tractors. A numerical rating system was designed that assigns a suitability score from three criteria, namely slope, road network proximity, and flood risk, based on established scales. Analytical Hierarchy Process was used as an approach to determine the relative influence in which results show that slope has the greatest weight (65%) followed by flood risk and road network proximity with 20% and 15%, respectively. The total suitability score (TSS) is then determined by getting the weighted average of the individual suitability scores. Land suitability mapping on the study area, the CALABARZON region, show that majority of the lowland rice areas are highly suitable for two-wheel and four-wheel tractors (81.39%) while some areas fall under marginal (15.03%) and moderate suitability (3.58%). Geotagged locations of the distributed agricultural tractors from 2015 to 2020 reveal that 78% of the distributed two-wheel tractors and 80% of four-wheel tractors in the region are situated in highly suitable areas. The GIS-based suitability model generated in this study can be utilized by the government to improve its machine distribution programs of two-wheel and four-wheel tractors in lowland rice farms.

## Introduction

The Philippines being an agriculture-based country largely cultivate rice since it is the staple food of Filipinos. It is being cultivated across over 4.8 million hectares in which 50% of the cultivated rice areas are low land ecology^1^. With the ongoing challenge of sustainable food production, the country’s agricultural machinery distribution programs for rice production have been strengthened over the past decade with about 39,000 Agricultural and Fisheries Machines (AFM) distributed from 2015 to 2020 which includes agricultural tractors, combine harvesters, and pump sets for irrigation designed for low land ecology. Moreover, about 36.4% of the total provision on agricultural equipment and facilities were allocated for rice production. Topping this with the additional annual Php 5 billion (approximately USD 90 million) allocation from the Rice Competitive Enhancement Fund (RCEF) program of the Philippine government^2^. In spite of the great efforts to boost rice production, concerns arose regarding the appropriateness of some of these distributed machines in which some of them became have not fully reached their economic life^3,4^. In 2018, the Philippine Council for Agriculture and Fisheries (PCAF) reported that 2 out of 10 (20%) of the distributed machines are either under-utilized or not utilized at all. Some instances include difficulties of machine transport from the distributors to the farms, or cases of waterlogged tractors from heavy flooding.

On the global perspective, Food and Agriculture Organization of the United Nations (FAO) has shifted its attention towards the promotion of sustainable agricultural mechanization. During the Global Conference on Sustainable Agricultural Mechanization (SAM) conducted in September 2023, notable advancement in SAM can be seen through the development of appropriate technologies such as tools, equipment, and machines to sustain crop production and land management. It is essential to expand and adapt these advancements in machinery usage to suit local land conditions or scaling up according to local land contexts to support SAM and to transform the agrifood systems and effectively manage land based natural resources. Additionally, to address the current global challenge on reliable access to safe, nourishing, and sufficient food, thereby contributing to achieve the United Nations’ Sustainable Development Goals (SDGs), especially SDGs 1 and 2^5^. These challenges and call for promoting SAM provided an avenue for experts to formulate policies that would improve machine distribution programs. Specifically, different studies on Decision Support Systems (DSS) for the selection of machines based on their technical specifications already exist in various literature^6,7^.

Using several criteria that are considered in the decision-making process, the use of Multiple Attribute Decision Making (MADM) approaches has been a useful tool to integrate sets criteria to determine an optimum alternative^8–10^. Literature review includes the use of a numerical rating system to rank different machines based on different technical and socio-economic factors^11–13^. Freeman and Ayers^14^ used NEBRASKA performance test results to rank different machines using a numerical rating scale of 0 to 10, while producing a database to collect the different rating of the machines for each identified technical criterion. Additionally, a critical value for each factor is set beforehand that would be used to screen the alternatives before being subjected to their own established numerical rating system of machine selection^15–17^. With the introduction of critical values, the number of alternatives that would be undergo the ranking system would decrease as the ones that do not meet the minimum value will not be evaluated anymore^15^.

Techniques in the formulation of rating systems for machine selection all involved the integration of identified factors. The simplest of which is by averaging the individual ratings of the factors to produce an overall rating^18^. This gives an assumption that all factors have the same influence in the overall objective. However, studies conducted by Lee and Chang^19^ and Wang et al.^20^ suggest that each criterion influences the objective differently, and their corresponding weight must be determined to achieve a more accurate ranking of alternatives. Several weighing methods are combined with MADM techniques on different machine selection schemes. The Analytical Hierarchy Process (AHP) is a widely used subjective weighing method which involves the translation of experts’ opinion through pairwise comparisons which then derives factor weights using matrix-based calculations^21^. Studies of Wang and Parkan^22^ and Xu^23^ added that the combination of the pairwise comparisons and matrix analysis in AHP takes into consideration the expertise of the decision makers while keeping bias at an acceptable level with the computation of the Consistency index (CI). To completely remove the bias from the determination of the weights, objective methods such as the Criteria Importance Through Intercriteria Correlation (CRITIC) approach uses standard deviation as basis to derive their relative weights^24,25^, and Shannon’s entropy method which determines the criterion weight by analyzing the probability distribution of the alternatives for each criterion^26,27^. Both methods; however, are highly data dependent because the presence of misfitting data such as outliers heavily affects the results.

In recent years, the combination of GIS and different MADM methods have been an effective tool in performing land suitability analysis on different aspects. Site suitability analysis is the most used in selecting the best location for the establishment of various structures. Asakereh et al.^28,29^ and Uyan^30^ used GIS and AHP-based suitability analysis approach in determining the optimum locations of solar farms given different environmental criteria while other studies imposed a numerical scoring system on site suitability analysis of different agricultural crops. In machine selection, AHP is used in studies conducted by Amini and Asoodar^31^ and Mehta et al.^32^ which involves ranking a set of machines based on performance criteria. Other objective MADM techniques like CRITIC and Shannon’s Entropy have also been used which in historical data were analyzed to determine the relative weights of performance-based factors.

Most literature only focused on the performance aspect of machine selection, but less attention has been directed towards the role of environmental factors on the appropriateness of machines. Studies conducted by Battiato and Diserens^33^ and Kim et al.^34^ investigated the effect of soil properties on the performance of agricultural tractors specifically on soil draft which is necessary for land preparation. Others focus on the identification of the optimal environmental conditions for the operation of machines. However, there is distinct lack on the application of geospatial data for suitability analysis of machines given that the environmental conditions of their respective farms affect their performance. This paper focused on the development of a GIS model for mapping appropriate agricultural tractors in lowland rice ecology using a numerical rating system using geospatial environmental criteria with AHP as the weighing tool.

## Materials and methods

**Figure 1:**
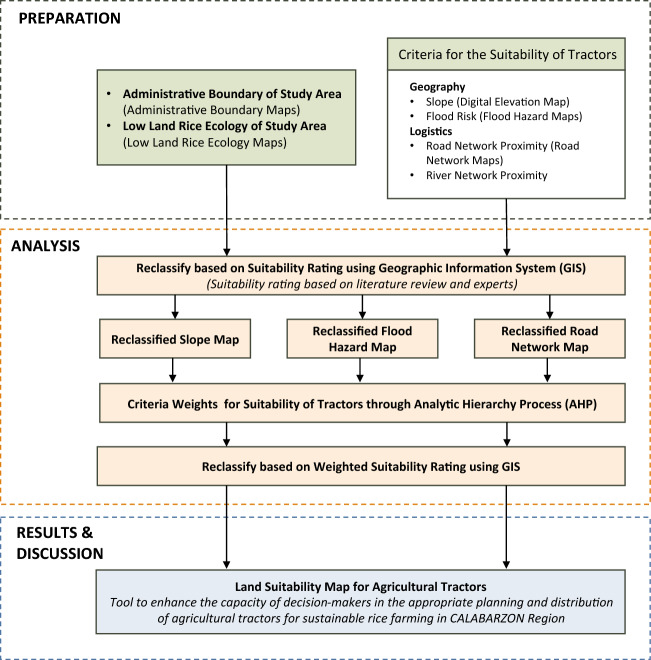
Process flow in the generation of land suitability maps for agricultural tractors in lowland rice ecology.

In this study, a GIS-based suitability model was developed for the evaluation of two-wheel and four-wheel tractors in lowland rice ecology. The first step was to identify the different environmental factors and constraints to be use in the proposed suitability model. Uyan^30^ used the maximum limitation method which identifies criteria, including terrain, geology, and distance to road networks, which affects agricultural output. This study used the mentioned method as basis of selection of environmental criteria. Three (3) different environmental criteria were considered, namely slope, road network proximity, and flood risk. These were identified based on literature review and experts’ opinion on the factors that affect optimum performance of agricultural tractors when situated in rice fields. Since the three criteria do not share equal influence on the production of the site suitability of agricultural tractors, the Analytical Hierarchy Process by Saaty^35^ was used. Finally, weighted overlay analysis method was used to integrate the spatial criteria to produce the land suitability map. The detailed process flow in the study is shown in Fig. 1.

### Study area

**Figure 2:**
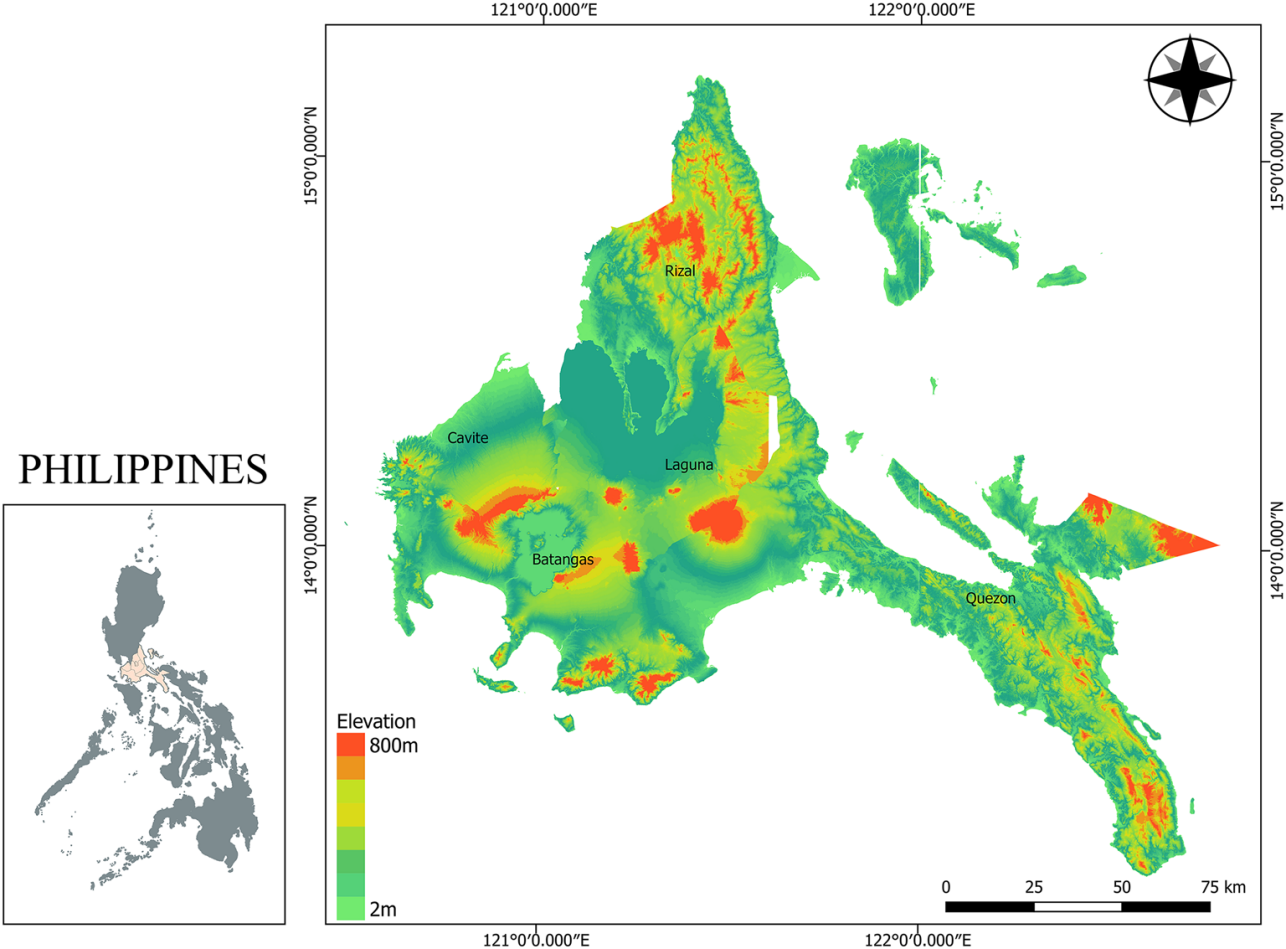
Elevation map of the study area. Figure was generated using QGIS 3.16 Hannover software (https://qgis.org/en/site/). *Data Source*: National Mapping and Resource Information Authority, Department of Environment and Natural Resources (NAMRIA, DENR) (https://www.namria.gov.ph/).

The area of interest is the *CALABARZON* region consisting of five provinces, namely Cavite, Laguna, Batangas, Rizal, and Quezon. It is geographically located about 14.1008° north of the equator and 121.0794° south of Luzon Island below the country’s National Capital Region (NCR). It has approximately 16,873 km^2^ of land area. The terrain of the region is both plain and hilly with elevations varying from 2 to 800 m as shown in Fig. 2*.* The Hilly regions of the study area include mountainous areas and volcanoes while residential and agricultural areas dominate the flat areas. Road networks connect the five provinces from the country’s National Capital Region which resides above the study area which makes the region a passageway for transit of market supply from the capitol to the southern parts of Luzon Island.

**Figure 3:**
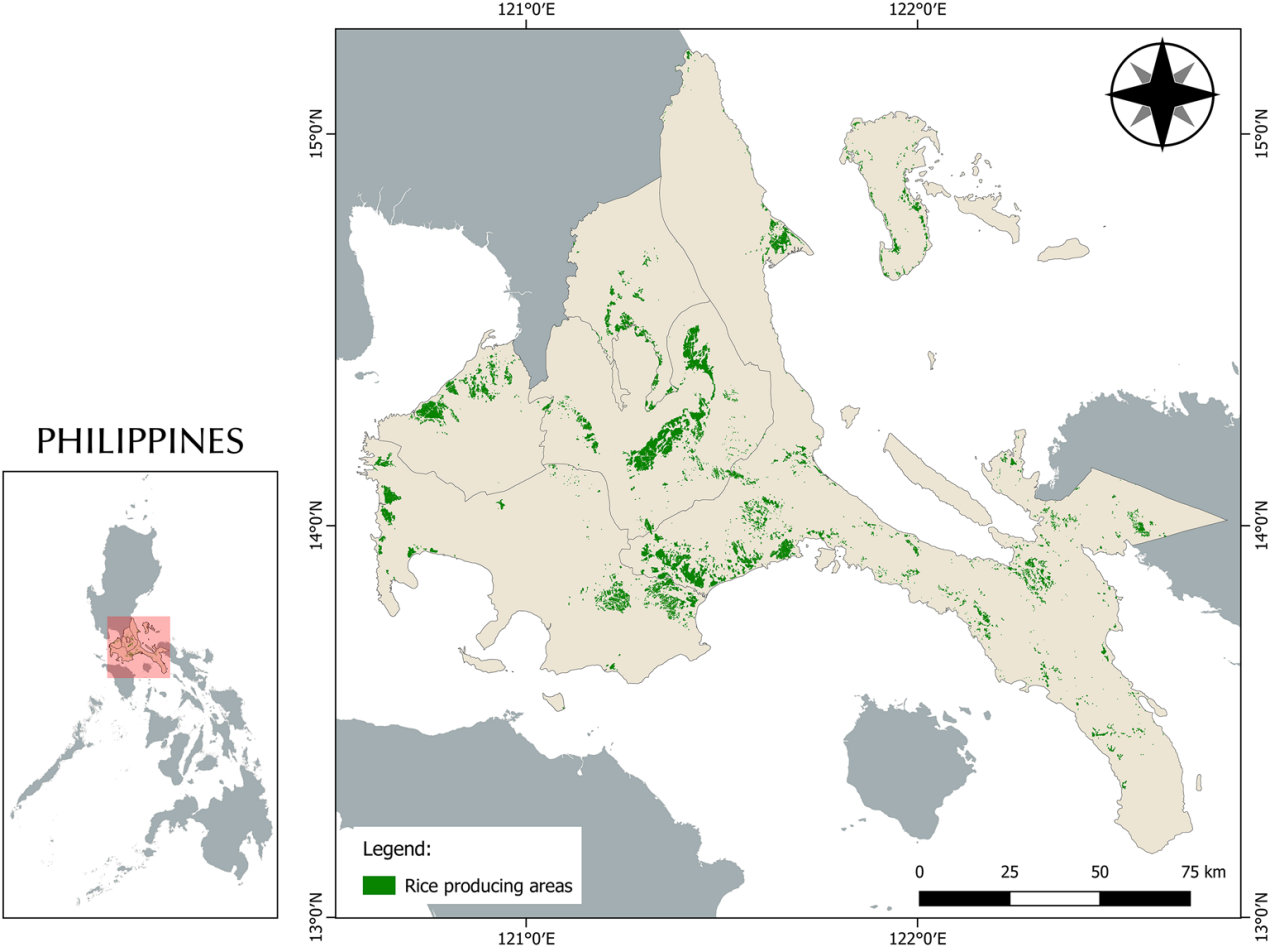
Rice producing areas of CALABARZON Region, Philippines. Figure was generated using QGIS 3.16 Hannover software (https://qgis.org/en/site/). *Data Source* Philippine Rice Information System, Philippine Rice Research Institute (PRiSM, PhilRice) (https://prism.philrice.gov.ph/).

The economic output of the region is mainly dependent with agriculture, rice being as one of the region’s largest commodities. According to the remote sensing data from the Philippine Rice Information System project (PRiSM) of the Philippine Rice Research Institute (PhilRice), lowland rice ecology of the region is approximately 54,443 ha (Fig. 3) with an average yield of 18.5 ton/ha as of 2021. Agricultural mechanization reports of the National Agri-fisheries Investment Audit Program of the country’s Department of Agriculture show that the *CALABARZON* region has received 2139 units of rice production machines or about 16% of the total machines distributed in the whole country from 2015 to 2020. The region ranked second in the overall audit of the total machines distributed for agricultural production (rice, corn, and Solar Powered Irrigation System (SPIS)) with a share of 17% of the total machines distributed.

### Generation of thematic maps for environmental criteria and constraints

The administrative boundary map of the study region sets the limitation to which the model would be performed. Also, suitability analysis is only limited on lowland rice ecology. The lowland rice farms were isolated using vector data provided by the Department of Agriculture—Philippine Rice Research Institute (DA-PhilRice) which were derived from C-band Synthetic Aperture Radar (SAR) images.

Slope has a significant influence on the performance of agricultural tractors as it critically affects the stability and maneuverability of agricultural tractors during field^36–38^. There exists a slope limit in which agricultural tractors can still efficiently operate for a certain horsepower rating. The basis of assigning the optimum slope range is based in a contiguous farm wherein Yoneyama et al.^39^ stipulated that a 65 hp tractor can only operate efficiently up to 8° slope. In this study, slope maps of the study region were generated using Quantum GIS (QGIS) software from Digital Elevation Maps (DEMs) obtained from the National Mapping and Resource Information Authority (NAMRIA), an agency that collects and analyzes geographic data in the Philippines. The DEMs have a resolution of 5 m and were derived from 2013 Interferometric Synthetic Aperture Radar (IFSAR) images.

Deployment and in-field transport of agricultural machines is a challenge especially if the farm is not connected to a well-defined road network system. Additionally, access to road networks links rice areas to nearby agricultural machinery service centers for tractor repairs and farm operation services. With this, road network proximity is considered as a factor in the determination of suitability of agricultural tractors. To obtain spatial road network information, a QGIS plugin called *QuickOSM* was used to access the OpenStreetMap (OSM) portal, an open-sourced database of geographical data. This study considered major road networks as well as Farm-to-Market Roads (FMRs) and other agricultural roads. Proximity analysis was then performed in QGIS to generate road network proximity maps.

Flood risk plays a role in keeping agricultural machines safe in cases of heavy rainfall such as the provision of machine sheds. The presence of these sheds is a requirement in the distribution of agricultural tractors in the Philippines. Assuming these machine sheds are built near rice farms, areas with high risk of flooding means that the tractors are more exposed to stagnant water causing damage which decreases its overall economic life^40^. Therefore, it is posited that this criterion is also a major factor that should be considered whether an agricultural tractor is suitable for a particular lowland rice farm. The 100-year flood risk maps of the study area were obtained from the Mines and Geosciences Bureau-IV-A (MGB IV-A). The different spatial datasets are summarized in Table 1, and all of which were processed using QGIS software.

**Table 1:** Datasets obtained by for the development of GIS model for the suitability of two-wheel and four-wheel tractors.

Spatial data	Description	Source
Administrative boundary	Locations of boundaries of Region IV-A the provincial level	NAMRIA
Digital elevation model	Digital Terrain Model (DTM) derived from 2013 airborne Interferometric Synthetic Aperture Radar (IFSAR) data in Universal Transverse Mercator Projection (UTM-Zone 51N) at 5 m resolution with the Philippine Reference System of 1992 (PRS92) as the horizontal datum	NAMRIA
Road networks	Location of Primary, secondary, tertiary, and agricultural roads derived from an open-source plugin in QGIS 3.10	OpenStreet Map
Rice planted areas	Locations of rice planted areas from 1st semester of 2018 to 2nd semester 2020 with a database of rice areas (hectares) in provincial level	PhilRice PRISM project
Flood hazard data	100-yr flood hazard maps	MGB
Locations of two-wheel and four-wheel tractors	Geotagged locations of two-wheel and four-wheel tractors distributed in Region IV-A from 2015 to 2020	DA Regional Field Office- IV-A

### Normalization of the thematic maps

Since the obtained thematic maps are expressed in different units, there is a need to normalize these spatial datasets so they can be mathematically integrated using weighted overlay analysis. In this study, a numerical scoring system was used to normalize thematic data into three suitability scores of 0, 1, and 2 corresponding to marginally, moderately, and highly suitable areas, respectively. A scale was developed containing the values of each criterion that corresponds to a suitability score. The suitability scales were formulated from an in-depth literature review, benchmarking of areas that employ field testing of agricultural tractors, as well as discussions with experts on agricultural machinery and mechanization.

According to the findings of Yoneyama et al.^39^, the most efficient performance of a 65 hp agricultural tractor in a contiguous farm is achieved on slopes less than 3°. This was used in this study as basis of the most suitable slope for agricultural tractors. Road network proximity affects the ease of in-field transport of agricultural tractors into rice farms and with the least logistical cost. This study proposes a buffer zone of < 100 m as the most suitable distance of the farm to the nearest road network. This zone gives the least distance in which tractors can be transported into the field with the least operational costs. Flood maps are usually categorized based on their level of risk, namely low, moderate, high, and very high flood risk. This was used a basis in the normalization of flood risk maps with low-risk and no-risk areas as the most suitable areas. The thematic maps were converted into raster format then were reclassified based on the established suitability scale. All layers are projected in the Universal Transverse Mercator (UTM) projection since metric units are present in the data.

### Determination of criteria weights

The Analytic Hierarchy Process (AHP) developed by Saaty^35^ is the most widely used Multiple Criteria Decision Making (MCDM) approach in land suitability studies because it considers experts’ opinion on criteria weighing while having data reliability through matrix-based calculations. Additionally, AHP has been used as weighing method in numerous literatures involving GIS-based suitability analysis on various applications^30,41,42^. With the use of AHP, the environmental criteria are laid out in a hierarchical structure wherein their percentage weight correspond to their relative significance with the overall objective^43^. These weights are evaluated by pairwise comparisons to derive a comparison matrix. This matrix consists of scores of 1–9 wherein a score of 9 indicates the highest significant of one criterion over the other as shown in Table 2. Matrix-based calculations are then performed to derive the weights. Afterwards, the reliability of the results is evaluated by the computation of the Consistency Ratio in which a value less than 0.1 is considered reliable, therefore the calculated weights are consistent with the evaluation of the decision makers with their bias kept at an acceptable level. Ten (10) experts on the field of agricultural machinery and mechanization from different government agencies, academe institutions, and machinery manufacturers participated in the survey for criteria weights determination using AHP.

**Table 2:** AHP scale for pairwise comparisons (Saaty^35^).

Importance rank	Definition	Interpretation
1	Equal importance	Two criteria influence equally to the objective
3	Low importance of one over the other	Judgement and experience slightly favor one criterion over the other
5	Strong or essential importance	Judgement and experience strongly favor one criterion over the other
7	Established importance	A criterion is strongly favored, and its dominance is established in practice
9	Absolute or high importance	The evidence favoring once criteria over the other is of the highest significance
2,4,6,8	Intermediate values	Adjustments between the other values

### Generation of land suitability maps

With the reclassified thematic maps in raster form and percentage weights derived from AHP, the Total Suitability Scores (TSS) were then computed using the weighted overlay as expressed in Eq. (1).

Formula for determining the total suitability score for agricultural tractors:

1 $$TSS= \sum_{i=1}^{n}{W}_{i}{S}_{i},$$

where *W*_*i*_ is the relative weight of the factor *i* derived from AHP, and *S*_*i*_ is the suitability score of the area based on that criterion. Lastly, the raster map containing the TSS of the study area was reclassified using the scale shown in Table 3. Map layout generation was created using QGIS software.

**Table 3:** Total Suitability Score (TSS) classification scale used in the land suitability model.

Total suitability score (TSS)	Classification	Description
< 0.66	Marginally Appropriate areas	Lowland rice production areas that are highly recommended for the distribution of two-wheel and four-wheel tractors
0.66–1.33	Moderately Appropriate Areas	Lowland rice production areas that are recommended for the distribution of four-wheel tractors, and highly recommended for two-wheel tractors
> 1.33	Highly Appropriate Areas	Lowland rice production areas that are not recommended for the distribution of four-wheel tractors but may be suitable for two-wheel tractors, provided that the farm or field is terraced

## Results and discussion

The identification of suitable lowland rice areas for two-wheel and four-wheel tractors was done in this study by integrating normalized spatial datasets of identified environmental criteria using weighted overlay analysis and AHP as weighing approach. The survey based AHP procedure on experts on agricultural machinery and mechanization revealed that slope comprises 65% of the suitability of tractors in lowland rice areas, followed by flood risk (20%), and road network proximity (15%). Four (4) levels of land suitability classification, namely highly suitable, moderately suitable, marginally suitable, and currently not suitable were used in the established scales to reclassify the thematic data. The four (4) classification categories were adopted from the Food and Agricultural Organization (FAO) which was also used by Pramanik^44^ on agricultural land suitability analysis. Since this study has already limited its suitability model to lowland rice ecology, only the first three (3) suitability classifications were the ones applied in the scale. The use of a three-level suitability scale is also used in other studies in land suitability classification especially on agricultural production such as rice implying that the use of three-level classification scale clearly assigns the significance of a piece of agricultural land in which a high suitability rating resembles ideal conditions while marginally suitable implies the opposite. The provision of moderately suitable areas adds an intermediate condition in which an area is neither ideal nor undesirable for an objective^45^.

**Figure 4:**
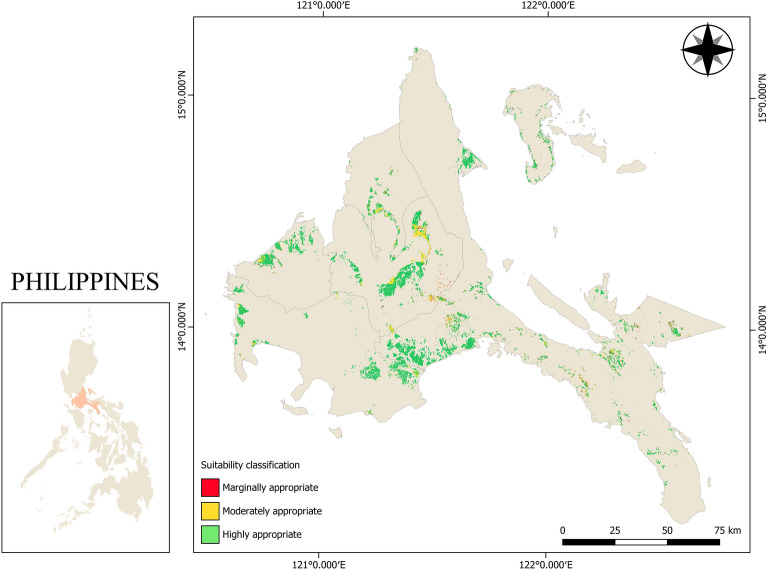
Land suitability map of CALABARZON region for agricultural tractors. Figure was generated using QGIS 3.16 Hannover software (https://qgis.org/en/site/). Data NAMRIA, DENR (https://www.namria.gov.ph/) and (PRiSM, PhilRice) (https://prism.philrice.gov.ph/).

**Figure 5:**
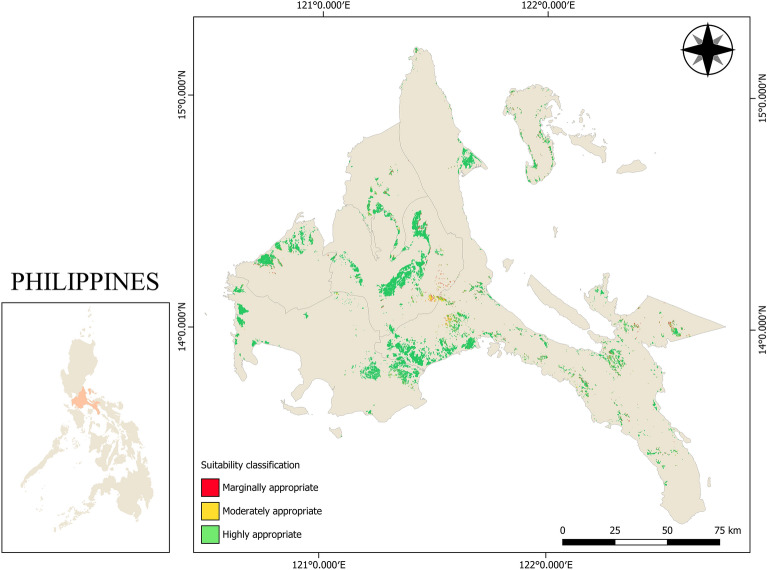
Slope suitability map of CALABRZON region. Figure was generated using QGIS 3.16 Hannover software (https://qgis.org/en/site/). Data NAMRIA, DENR (https://www.namria.gov.ph/) and (PRiSM, PhilRice) (https://prism.philrice.gov.ph/).

With 65% criterion weight, the large influence of slope on the selection of mechanization technologies such as tractors is the effect of terrain on the operation of agricultural machinery. A study conducted by Akinci et al.^46^ revealed that slope has the highest contributed weight on the selection of land for agricultural use when AHP was used as weighing method. Yang et al.^47^ studied the land suitability of agricultural machines on consolidated lands in which 15 different assessment indicators were considered and machine learning approach was utilized to allocate weights of the indicators. Their study showed that among the 15 indicators, slope has the significant influence on land suitability for agricultural machines. Cogato et al.^48^ also conducted a study on the potential mechanizability of vineyard areas in which seven (7) parameters were considered. Their research indicated that slope is the main contributing factor on accessibility of production areas for agricultural machines. Similarly, this study showed that slope was the critical influence for land suitability of agricultural tractors as seen in Table 4. The suitability scale used in this study stated that marginally appropriate areas only allow the use of two-wheel tractors in lowland rice areas if the area is terraced because on a steeply gradient farm, the operation of four-wheel tractors will not be fully implemented since the terrain will cause the four-wheel tractors to topple, causing damage to both the machine and its operator even with the provision of Roll-Over Protection Structures (ROPS) and four-wheel drives. With this, the suitability model says that if the slope of the rice farm is greater than 8°, only the provision of two-wheel tractors is allowed with the additional requirement that the rice farm is in a terraced area. Alternatively, farms whose terrain is less than 3° are most recommended for both two-wheel and four-wheel tractors. The effect of having a large percentage weight for slope is clearly shown on the comparison of the overall land suitability map in Fig. 4 and slope suitability map in Fig. 5 using the established suitability scale. The similarities with the two maps show that a huge consideration in the selection of two-wheel and four-wheel tractors is based on the terrain of the rice area. Considerably, areas that fall under marginal suitability should undergo site verification if the rice field is terraced for it to be suitable for two-wheel tractors.

**Table 4:** Suitability scales and weights of the criteria considered in the study.

Criterion	Weight (%)	Range of values	Suitability score*	Consistency index
Slope	65	< 3°	2	0.06
		3°–8°	1	
		> 8°	0	
Road network proximity	15	< 100 m	2	
		100–200 m	1	
		> 200 m	0	
Flood risk	20	Low flood risk	2	
		Moderate flood risk	1	
		High to very high flood risk	2	

*2—highly suitable areas, 1—moderately suitable areas, 0—marginally appropriate areas.

**Figure 6:**
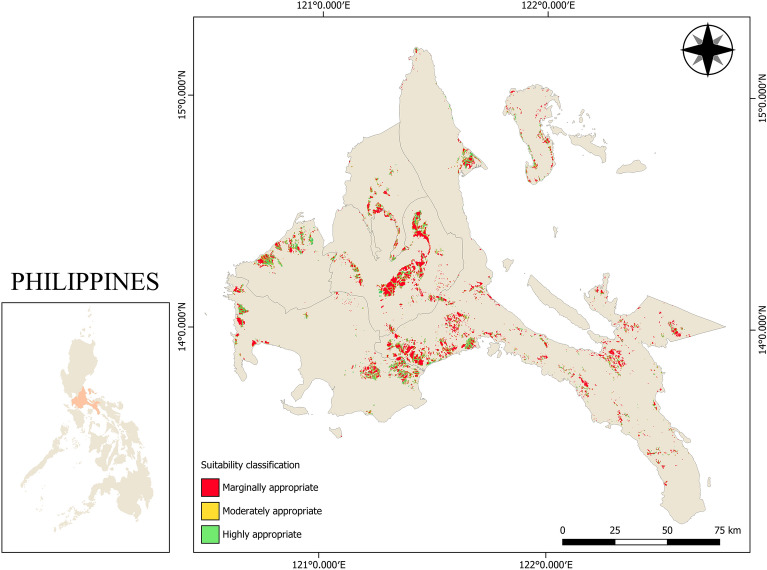
Road network proximity suitability map of CALABARZON region. Figure was generated using QGIS 3.16 Hannover software (https://qgis.org/en/site/). Data NAMRIA, DENR (https://www.namria.gov.ph/) and (PRiSM, PhilRice) (https://prism.philrice.gov.ph/).

The importance of road network proximity has been a key discussion in multiple studies concerning sustainable food production. Though this criterion constitutes the smallest percentage AHP-derived weight for the suitability of agricultural tractors, its significance should not be understated. A comprehensive study done by Barbour^49^ that harvest quality among perennial and annual crops, including rice, partly depends on how close their farms are to road systems connecting to processing centers or marketplaces. Petit et al.^50^ survey instruments and policy reviews of the Controlled Resources and Crops (CRC) guidelines of France to evaluate the impact of farm-to-market roads to farmer and retailers of different agricultural commodities. Isolation distances, defined in their study as clear pathways 50 m to 200 m from the farm to the road system were identified as the range in which annual and perennial crops will be kept at best quality upon transport from the farm to processing centers. This study adopts such values with a specific premise that agricultural machines will be transported easily from their sheds to their respective farms efficiently without costing excess fuel and time on the farmer. Figure 6 shows the road network proximity map and it is clearly shown that majority of the rice areas are situated farther than 200 m from the nearest road network, which corresponds to marginal suitability. However, the effect of the road network suitability does not greatly influence the overall land suitability map because of its low percentage weight. This means that logistics costs including in-field transport of tractors is highly compromised in the evaluation suitability for lowland rice ecology as compared with slope. Having rice areas at high risk of flooding affects the economic life of agricultural machines such as tractors**.**

**Figure 7:**
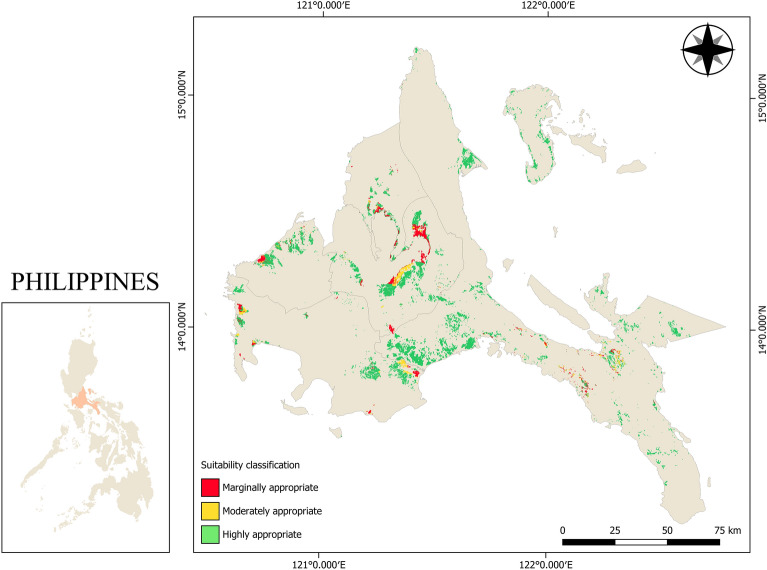
Flood risk suitability map of CALABARZON region. Figure was generated using QGIS 3.16 Hannover software (https://qgis.org/en/site/). Data NAMRIA, DENR (https://www.namria.gov.ph/) and (PRiSM, PhilRice) (https://prism.philrice.gov.ph/).

A key parameter that was uniquely considered in this study was the flood risk, as flooding is a prominent issue in the Philippines, which would greatly affect the usability and life of agricultural tractors. A study by Posthumus et al.^51^ pointed out that damages on agricultural machinery due to flooding is considered as direct instantaneous damage because the damages immediately decrease the usability and economic life on a machine while Erdlenbruch et al.^52^ pointed out that damaged machines are a primary cause of delay on farm recovery because it lessens the time for harvesting crops that were supposed to be salvaged after natural disasters, as well as delays on conducting land clearing before the start of a new planting season. It is therefore stated that risk management towards putting the correct machines in the field is necessary to avoid unnecessary damage and to increase productivity. Since this study assumes that machine sheds are placed near the farms they are operated upon, the role of having suitability criteria for flood risk is also a huge factor on the selection of suitable two-wheel and four-wheel tractors in lowland rice ecology. Since flood risk does not differ in road network proximity in terms of percentage weight derived from AHP, the same concept can be said that the presence of rice areas that have moderate to high flood risk may not be clearly represented in the overall suitability for tractors as shown in Fig. 7.

The resulting map (Fig. 4) showed the different levels of for agricultural tractor operations. The land suitability map was divided into three (3) suitability classifications: marginally appropriate, moderately appropriate, and highly appropriate. A significant portion of the study area, 81.39% (44,311 ha), is highly appropriate for agricultural tractors, both two-wheel and four-wheel tractors. These areas are mainly located near bodies of water for continuous rice production and at lower elevation for the maneuverability of tractors. About 15.03% (8166 ha) of the study area is found to be moderately suitable for agricultural tractors. These areas are mainly located in Laguna and Quezon Provinces of the study area. The marginally appropriate area for agricultural tractors covers only 3.58% (1949 ha), which are concentrated in Laguna and Rizal Provinces of the study area.

The difference with the percentage weight of the three (3) criteria means that one (1) criterion must be compromised with the others. In this study, slope was found out to be the greatest priority on the determination of suitability of lowland rice farms for agricultural tractors while distance to road networks being the least. Having these results, the authors suggest that the result of AHP implies that those factors with lower percentage weight can be compensated easier than those with higher percentage weights. For instance, road network proximity of farms in marginally appropriate areas can be improved with the establishment of Farm-to-Market Roads (FMRs) which can be a good investment for future farm activities, as compared with terracing rice farms which requires higher logistics and operational costs. Furthermore, the conduct of cut-and-fill operations delays rice production in concerned farms which affects rice yield among farmers. Additionally, it is easier to construct machine sheds on higher ground proximate to the farms to prevent the machines from being damaged as Erdlenbruch et al.^52^ mentioned that damage valuation is much more expensive than having prevention measures for agricultural machines. The said concept is valid in this study as the construction of FMRs and machines sheds to improve the ease of operation and economic life of agricultural tractors are cheaper than having them replaced every five (5) years or less because of flooded engines or parts replacement. These preventive measures for road network proximity and flood risk are seen to be better alternatives than to compensate for slope suitability as seen in the results of the AHP-based suitability weight analysis. Therefore, preventive adjustments to improve road network proximity and flood risk of the lowland rice farms are ways to compensate their low relative weights in the suitability model*.*

The model constructed in this study has great advantage of considering the criteria based on several individual research^46–48,51,52^. The weights of each criterion were determined through AHP conducted with the experts in the field of agricultural machinery. Accordingly, it is a summative presentation of the key elements in the determination of suitable areas in lowland rice ecology for tractors. Moreover, the inclusion of flood risk criteria in the model addresses the flood-prone areas for growing rice in the Philippines to enhance the economic life and usability of the machine as the country is positioned within the typhoon-prone Pacific region which encounters approximately 20 typhoons annually, making it susceptible to flooding^53^.

**Figure 8:**
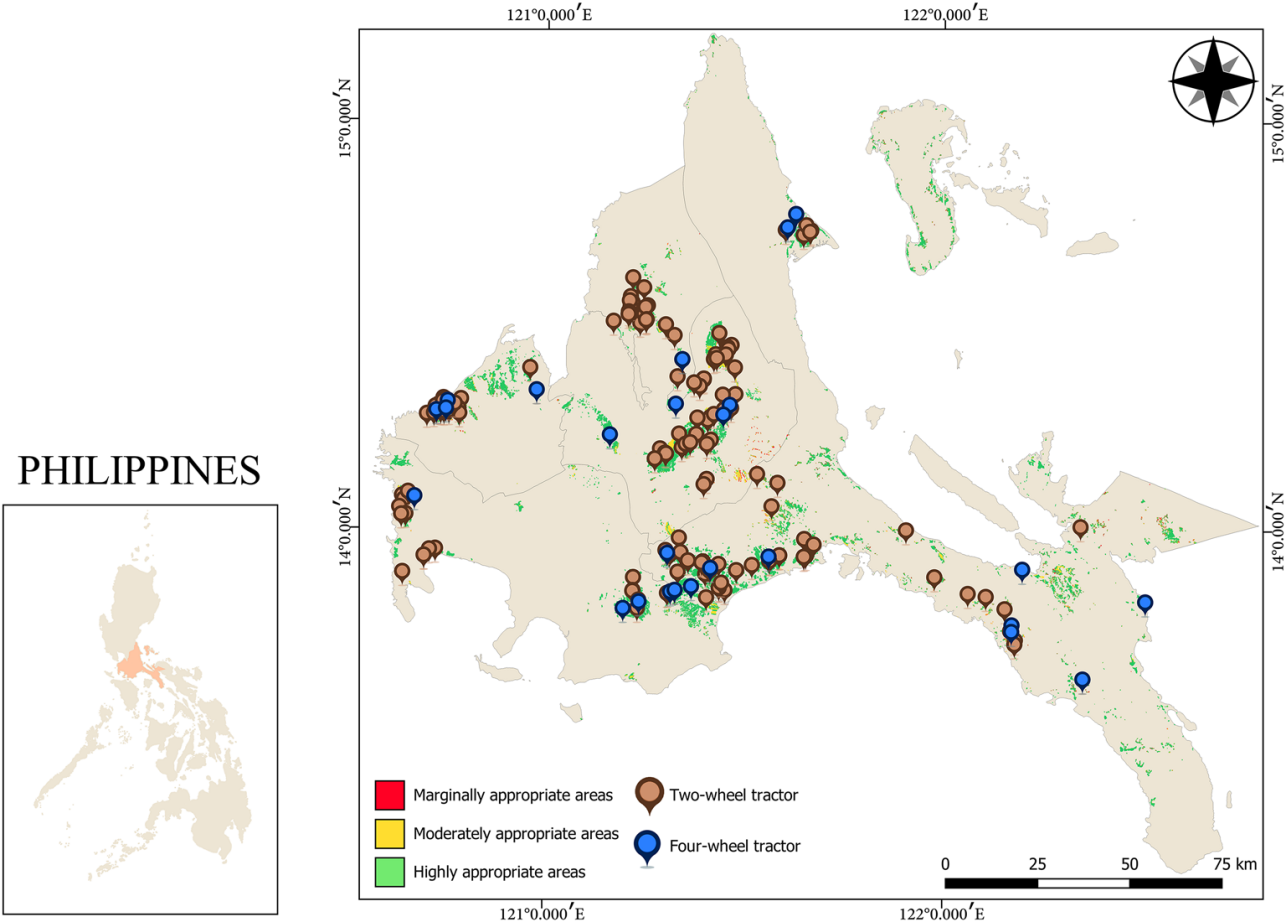
Distributed agricultural tractors in CALABARZON region. Figure was generated using QGIS 3.16 Hannover software (https://qgis.org/en/site/). Data NAMRIA, DENR (https://www.namria.gov.ph/) and (PRiSM, PhilRice) (https://prism.philrice.gov.ph/).

Verification of the model was done by collecting georeferenced locations of the distributed rice agricultural tractors in *CALABARZON* region shown in Fig. 8 from 2015 to 2020 and overlaying them in the generated land suitability map. It was found out that most of the available two-wheel (78%), and four-wheel (80%) tractors are situated in highly appropriate areas. While 17% of two-wheel and 4% of four-wheel tractors are found in moderately appropriate areas. However, the model shows seven (7) two-wheel tractor units and four (4) four-wheel tractor units that are located in marginally appropriate areas as summarized in Table 5. Following the suitability scale in this study, the farms containing the seven (7) two-wheel tractors are located in a terraced area (with the need of site verification), otherwise, the model dictates that those units located in marginally appropriate areas are needed to be validated through field experiments to see if they are fit to be in their respective rice farms.

**Table 5:** Suitability of distributed agricultural tractors in CALABARZON region.

	2-Wheel tractors		4-Wheel tractors	
	Units	%	Units	%
Marginally appropriate areas	7	4.67	4	16
Moderately appropriate areas	26	17.33	1	4
Highly appropriate areas	117	78	20	80

The results of the verification run using the established model for the determination of suitable areas for agricultural tractors showed that most of the distributed agricultural tractors in *CALABARZON* Region were located in highly appropriate areas. This could serve as a basis in establishing the land suitability map of tractors distributed in the other regions of the Philippines. Moreover, decision makers can use the land suitability maps to decide whether to put two-wheel or four-wheel tractors on a particular rice area based on their spatial characteristics. Additionally, the suitability maps for each criterion are also tools in formulating actions to improve the suitability of rice farms for agricultural tractors and establish land management approaches, guided by the land's physical characteristics. For instance, road network suitability maps can be viewed as an evaluation tool for the construction of Farm-to-Market Roads (FMR) connecting the rice farms to the different road network systems. From the research outputs of Petit et al.^50^ it can be stated that the addition of FMRs connecting the farms to highways with a strict buffer of 50 m to 200 m will improve the quality of harvest as well as protect the condition of machines as the provision on concrete roads will protect the tractors from damage as they bring the harvest to post-production facilities. The suitability maps can also be used as validation tool to locate specific points on where to put machine sheds such that multiple tractors can be accommodated which will protect them from flooding.

## Conclusion and recommendation

Agricultural machinery distribution programs in the Philippines have been an answer to the call of sustainable rice production, and these can be improved by strengthening their operational schemes. This paper proposed a suitability model of selecting agricultural tractors in lowland rice ecology from three environmental criteria, and suitability scores as bases of classification. Weighing analysis using AHP revealed that slope had the highest percentage weight among the three environmental criteria which explains the huge effect on the overall land suitability map. The use of a three-level suitability classification scale allowed the model to determine the areas in which only two-wheel tractors are allowed, and areas in which both two-wheel and four-wheel tractors are highly recommended. However, with the inspection of the individual suitability maps of the three criteria, it was found out that because of the differences in percentage weight, some factors were compromised over the others, especially road network proximity in terms of suitability. In an in-depth analysis of various literature, the proximity of farms to road networks affects the quality of produce for annual and perennial crops such as rice and affects operational costs such as fuel cost. Validation of the suitability model was performed by overlaying geotagged locations of the distributed agricultural tractors in the study area, and while majority are in highly suitable areas, some were still located in marginally appropriate areas which may implicate that they might not be efficiently utilized in their corresponding farms. The developed land suitability map of agricultural tractors from this study would be beneficial both for the decision-makers and end-users. It is a tool that can enhance the capacity of decision-makers to strategically allocate funds and provisions on the distribution of appropriate and locations-specific agricultural tractors for the sustainability of the rice production system. Moreover, policy makers can recommend schemes on proper recall and redistribution of agricultural tractors which were found to be situated in marginally suitable areas. For end-users, appropriate agricultural tractors could improve the timeliness of farm operations and could lessen the problems encountered in utilizing agricultural tractors thereby improving rice production to face issues of food security and uplift the lives of farmers.

However, the study has limitations and to improve the results for future studies, the authors suggest on exploring different ways on determining the relative weights of the criteria, specifically numerical methods or machine learning approaches that determine the influence of the factors based on ground data, which will be free from subjectivity. Additionally, since this study is limited only on the environmental aspect of agricultural tractor suitability, it is recommended to extend the study on the technical and socio-economic aspects like the development of a scheme on machine selection based on historical performance test results and consider indicators such as labor situation, farmer’s income, and age of agricultural tractors. Soil properties, and farm shape and size should also be considered as an indicator for assessment to identify appropriate size (in hp) of agricultural tractors in a lowland rice ecology. Proximity to agricultural tractor service centers would also be an influencing factor, especially on the sustainability of agricultural tractors. Development of operational policies among the selection of machines such as the provisions of farm shed and FMRs are recommended by the authors as these will further improve the suitability of the lowland rice farms that will fully utilize the distributed agricultural tractors. With a more in-depth analysis on the determination of suitability of agricultural machines based on other aspects aside from the environmental ones, the selection, procurement, and distribution of these machines would be more effective.

## Data Availability

The datasets used and/or analysed during the current study available from the corresponding author on reasonable request.
